# The capsaicin receptor TRPV1 is the first line defense protecting from acute non damaging heat: a translational approach

**DOI:** 10.1186/s12967-019-02200-2

**Published:** 2020-01-17

**Authors:** Daniela C. Rosenberger, Uta Binzen, Rolf-Detlef Treede, Wolfgang Greffrath

**Affiliations:** 1 Department of Neurophysiology, Mannheim Center for Translational Neuroscience (MCTN), Heidelberg University, Ludolf-Krehl-Straße 13-17, 68167 Mannheim, Germany; 2 Department of Cardiovascular Physiology, European Center for Angioscience (ECAS), Medical Faculty Mannheim, Heidelberg University, Mannheim, Germany

**Keywords:** Near infrared laser, Heat pain, Nociception, Desensitization, Primary sensory neurons

## Abstract

**Background:**

Pain is the vital sense preventing tissue damage by harmful noxious stimuli. The capsaicin receptor TRPV1 is activated by noxious temperatures, however, acute heat pain is only marginally affected in mice after TRPV1 knockout but completely eliminated in mice lacking TRPV1 positive fibers. Exploring contribution of candidate signal transduction mechanisms to heat pain in humans needs translational models.

**Methods:**

We used focused, non-damaging, short near-infrared laser heat stimuli (wavelength 1470/1475 nm) to study the involvement of TRPV1-expressing nerve fibers in the encoding of heat pain intensity. Human psychophysics (both sexes) were compared to calcium transients in native rat DRG neurons and heterologously expressing HEK293 cells.

**Results:**

Heating of dermal and epidermal nerve fibers in humans with laser stimuli of ≥ 2.5 mJ (≥ 25 ms, 100 mW) induced pain that increased linearly as a function of stimulus intensity in double logarithmic space across two orders of magnitude and was completely abolished by desensitization using topical capsaicin. In DRG neurons and TRPV1-expressing HEK cells, heat sensitivity was restricted to capsaicin sensitive cells. Strength duration curves (2–10 ms range) and thresholds (DRGs 0.56 mJ, HEK cells 0.52 mJ) were nearly identical. Tachyphylaxis upon repetitive stimulation occurred in HEK cells (54%), DRGs (59%), and humans (25%).

**Conclusion:**

TRPV1-expressing nociceptors encode transient non-damaging heat pain in humans, thermal gating of TRPV1 is similar in HEK cells and DRG neurons, and TRPV1 tachyphylaxis is an important modulator of heat pain sensitivity. These findings suggest that TRPV1 expressed in dermal and epidermal populations of nociceptors serves as first line defense against heat injury.

## Introduction

Sensation of pain is maybe the most important sense *quoad vitam* since it protects against injury and tissue damage by harmful stimuli. Molecular sensors detecting thermal or mechanical tissue damaging stimuli, however, still remain elusive; particularly those detecting threat and not actual damage—the first line of defense. Translation of findings from one system level (in vitro) to another (in vivo) and finally to humans is one of the great challenges in medical science in general [15], as direct translatability of methods and tools, applicable at more than one system level, is often difficult [61]. We now investigated intensity coding of the same adequate stimulus at different system levels, facilitating translation of peripheral encoding of noxious heat pain from cellular models to human experiments using a specific non-damaging noxious heat stimulus.

The capsaicin receptor TRPV1 has been described as a cation channel gated by noxious heat [10]. Phosphorylation by different kinases (PKA, PKC, MAPK), mirrors peripheral sensitization to heat by inflammatory mediators [25] and the resulting primary hyperalgesia following injury [9, 10, 13]. The competitive TRPV1 antagonist capsazepine (CPZ) inhibits heat-induced inward currents in TRPV1-expressing cells [10] and nociceptive neurons [31, 34]. While mice with depleted TRPV1 carrying nerve fibers selectively lose heat sensitivity [11], TRPV1 knockout mice still have behavioral sensitivity to ramped heat stimuli [9, 13]. Two additional TRP channels, TRPM3 and TRPA1, seem to contribute to the perception of heat pain [60]. The lack of an acute heat phenotype when knocking out TRPV1 may be explained by absence of functional relevance for acute pain or by mismatch of heat stimulus characteristics and TRPV1 thermal gating properties. Until now, there is a mismatch of stimulation paradigms between recent molecular, electrophysiological and behavioural studies of heat pain. Contact heat stimuli are applied by thermodes in human and animal studies [12, 16, 60], while at the cellular and molecular level superfusion with heated solutions is used [52, 60]. Radiant heat stimuli that allow precise control of stimulus timing, are considered gold standard for clinical electrophysiological studies of heat pain pathways [23] but are rarely used in vitro [19, 27, 64]. Infrared lasers have been used in a few studies on animal behaviour [1, 8, 39, 63], electrophysiology [14, 56, 58], dorsal root ganglion (DRG) neurons [19, 40] and heterologously expressed TRPV1 [27, 64]. Wavelengths of these lasers, however, range between 980 and 10,600 nm [19, 59], spot sizes between 0.1 and 10 mm [27, 41] and pulse durations between 3 and 400 ms [19, 53], making comparisons across studies difficult.

The aim of this study was to facilitate the direct translation of peripheral encoding of noxious heat from cellular models to human experiments using the same near-infrared laser stimuli in three system levels. Encoding properties of transient, non-damaging laser heat stimuli will be characterized in human psychophysics and rat DRG neurons and compared to heterologously transfected HEK293 cells. Involvement of TRPV1 in signal transduction will be compared between native neurons and a heterologous expression system regarding thresholds, suprathreshold encoding, strength-duration curves and tachyphylaxis.

## Materials and methods

**Fig. 1:**
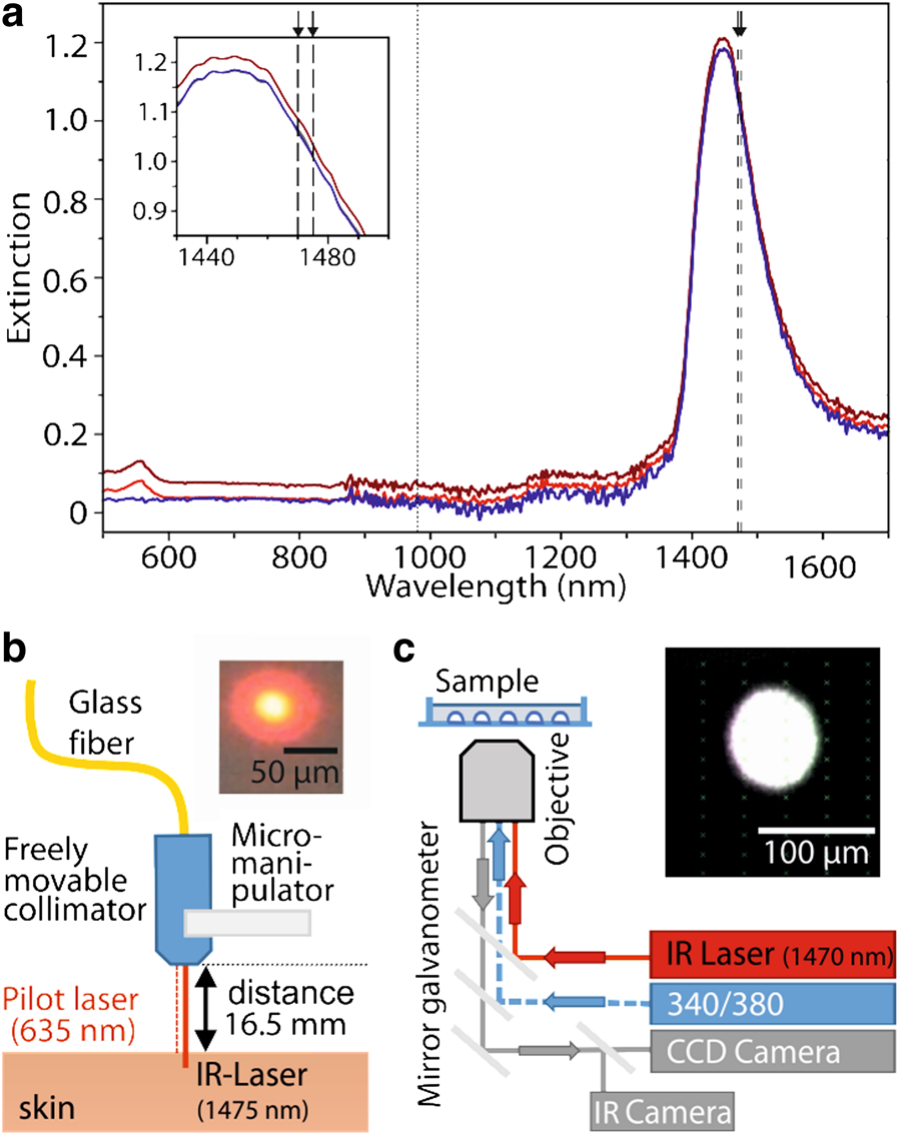
Setups for diode laser heat stimulation in vivo and in vitro. **a** Absorption spectra of water (blue), DMEM F12 medium (red) and DRG neurons suspended in DMEM F12 medium (dark red). DMEM F12 with-/without neurons had an absorption peak near 550 nm due to phenol red, suspended neurons induced a slight parallel upwards shift of absorption spectrum due to scattering. Wavelengths of our two diode lasers near the absorption peak of water (1450 nm) are indicated as dashed lines. Inset shows the almost identical absorption of the slightly different wavelengths used (arrows). Wavelength of a laser used previously is marked with dotted line [19]. **b** Schematic view of set-up for studies in humans. A pilot laser (635 nm) is used for aiming at the skin surface (see inset); the 1475 nm laser radiation has its focus 930 µm below skin surface. **c** Schematic view of integrated laser stimulation set-up for cell physiology experiments. The diode laser (DL-1470; “IR Laser”) is coupled into an inverted microscope via a beam splitter and focused to the image plane of the microscope. Mirror galvanometers move the laser beam to excite cells directly without any absorbance by aqueous solution. Aiming is verified by an infrared camera on the microscope (see inset)

Two diode lasers were used with wavelengths near an absorption peak in water and thus approximately in skin (λ = 1470 nm/1475 nm; see Fig. 1a; [43, 64]). Both lasers were focused to a small diameter (nominally 100 or 150 µm), allowing for rapid passive cooling and selective activation of single cells on a microscope cover slip as only a very small volume is heated. Extinction spectra of DRG neurons in F12 medium were measured in 10 mm quartz glass cuvettes from 500 to 2200 nm in triplicates, using an Excalibur Series FTS 3000 Infrared Spectrometer (BioRad, Munich, Germany).

### In vivo model: humans

#### Diode laser stimulation in humans

On hairy skin of the dorsal side of hands and feet heat stimuli were applied with a focused diode laser stimulator with a nominal maximum power of 100 mW, and a diameter of 150 µm on skin surface at 1475 nm (Laserdioden-Strahlquelle SK9-2001; Schäfter + Kirchhoff, Hamburg, Germany). An integrated pilot laser with a wavelength of 635 nm was used for focusing by keeping the distance between fibre tip and skin constant; thereby maximum heating of skin layers below surface was achieved (see Fig. 1b).

**Fig. 2:**
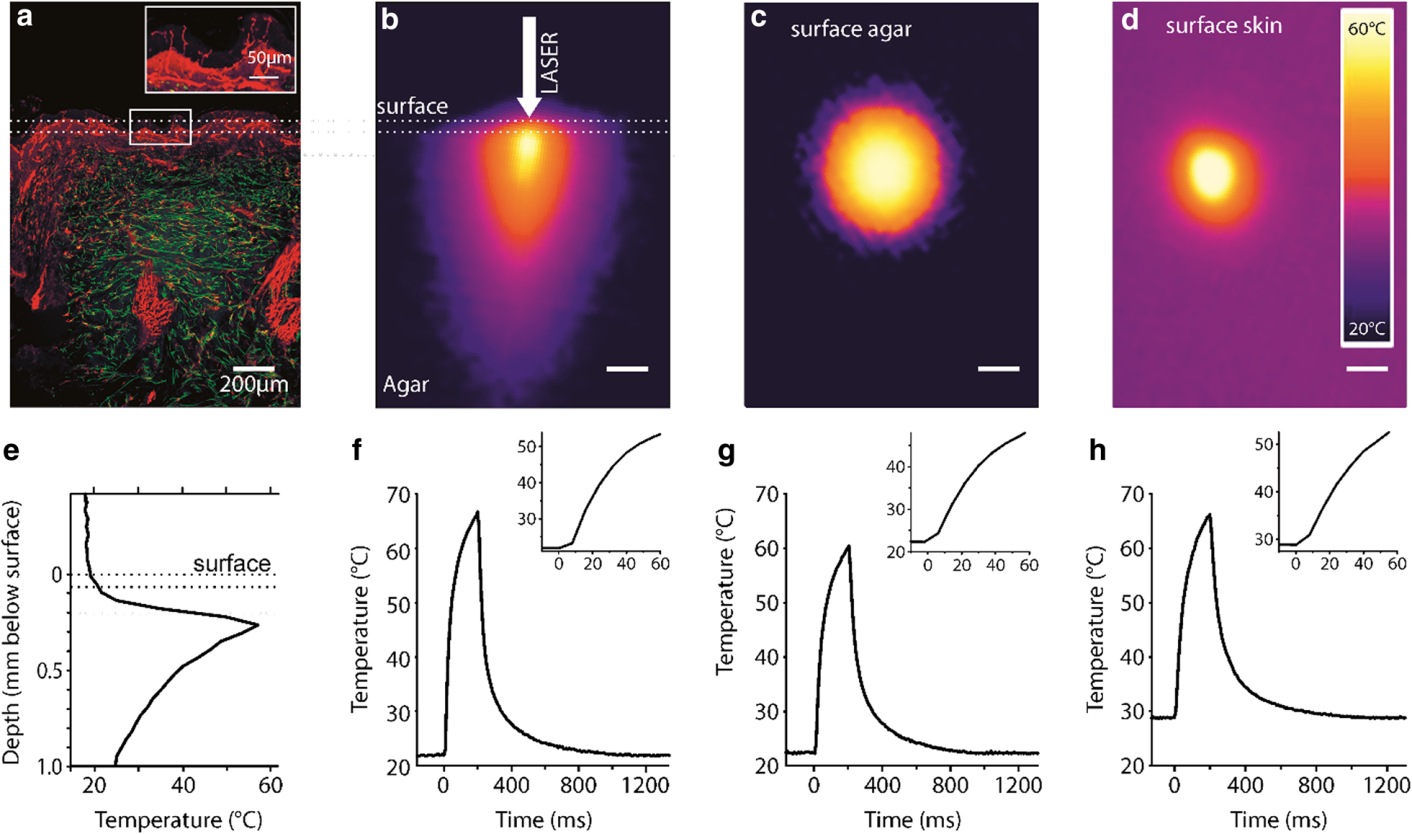
Focused diode laser stimuli rapidly increase temperature within a small volume of human skin. **a** Histology of human skin stained for the neuronal marker PGP9.5 to display free epidermal and dermal nerve endings in red color (skin biopsy kindly provided by Claudia Sommer, Würzburg). Dashed lines indicate approximatively borders of epidermis/dermis and dermis to facilitate comparison to agar model in (**b**). Insert shows intraepidermal fibers at higher resolution. **b** Spatial distribution of temperature changes with a Gaussian distribution as a consequence of a laser stimulus (100 mW, 200 ms) using agar as a model of human skin (side view with thermocamera Optris PI 640). The heated volume is small and the maximum heating is achieved at about 280 µm depth. **c** Temperature distribution on the surface of the agar model and **d** on the skin surface of the human hand dorsum. **e** Thermoprofile of (**b**) confirming the maximum temperature at about 280 µm below surface, dotted lines as in (**a**, **b**). **f**–**h** Time courses of temperature changes due to a single laser stimulus obtained from the experiments shown in (**b**–**d**), inset in (**f**–**h**) display magnification to visualize threshold temperatures. Scale bars in (**a**–**d**) 200 µm

The laser used in humans in vivo was focused to 930 µm below skin surface, using a difference in focal length between the visible pilot laser (focused on the skin surface to indicate the stimulus location; see inset in Fig. 1b) and the near infrared stimulation laser to heat the epidermis and dermis (Fig. 2a). To characterize the steep ramped laser stimuli in humans, thermographic measurements of the effective temperature were performed using an infrared thermocamera (PI 640 with microscope optics, Optris, Berlin, Germany) with Optris PI Connect software (Rel. 2.3) at an acquisition frame rate of 125 Hz. We recorded from the surface of human skin on the dorsal hand, as well as surface and side view (Fig. 2b–d) of a 4% agar model for skin [47] to monitor temperature changes at deeper layers in Z-direction (Fig. 2e) and to determine temporal stimulus characteristics (Fig. 2f–h). Laser heat profiles developed according to the Gaussian beam profile (Fig. 2b–d) and temperature maxima were about 280 µm below surface (Fig. 2b, e). Additionally to the intraepidermal nerve fibers that are evaluated clinically, there is also a dense network of nerve fibers within this depth range (insert in Fig. 2a). Temperature rose nearly exponentially at the surface of the skin (Fig. 2h), the surface of an agar phantom (Fig. 2g) and at 280 µm below the agar surface (up to 320 °C s^−1^; Fig. 2f). Passive cooling was exponential with a time constant of τ = 0.35 ± 0.01 s on skin, 0.29 ± 0.01 s on agar and 0.32 ± 0.01 s beneath surface (Fig. 2f–h). No heat accumulation was measurable for interstimulus intervals (ISIs) ≥ 1 s. Peak temperature increase was 37.8 ± 0.5 °C above baseline (Fig. 2f) for this strong suprathreshold stimulus (200 ms, 100 mW). Baseline temperature in hands was 29.8 ± 0.5 °C, in feet 26.7 ± 1.2 °C.

#### Human psychophysics

Experiments were performed in healthy human volunteers (n = 10; 3 female, 7 males, age 21–30 years, 25.7 ± 2.5 mean ± SD). All volunteers gave written informed consent after careful explanation of the nature and possible consequences of the studies; the experiments were approved by the local ethics committee. Laser pulses of different durations (range: 5–390 ms, at least 5 stimuli per duration and side) were applied at constant power of 100 mW to the dorsum of the hands and feet. Even with maximum duration (390 ms) no side effects other than small, slight and transient red spots were observed, particularly no burn injury. Painfulness was assessed using a numeric rating scale (NRS) ranging from 0 (= no pain) to 100 (= strongest imaginable pain). Pain thresholds were determined as the minimum stimulation duration inducing a painful hot or pinprick-like sensation, using a staircase regime (method of limits). The site of stimulation was slightly changed after each stimulus in order to avoid nociceptor suppression and tachyphylaxis of *I*_heat_ [33, 52]. To quantify tachyphylaxis, in separate experiments the same spot was stimulated repeatedly [17] at an interstimulus interval (ISI) of 180 s like in the in vitro experiments.

#### Desensitization of TRPV1-expressing nerve fibers

Adhesive skin patches containing 8% capsaicin (Qutenza^®^, Astellas Pharma, Munich, Germany) were applied on the dorsal side of one hand and one foot for 24 h in an area of about 3 × 3 cm to reversibly desensitize TRPV1-expressing nerve terminals in the skin [24]. On the contralateral area, a vehicle patch without capsaicin was used as a control (Qutenza^®^ Demo, Astellas). Numeric pain ratings and threshold determination in test and control areas were compared before and after application of the patches.

#### Data analyses

All NRS ratings were log_10_-transformed after addition of a constant of 0.1 to avoid loss of zero ratings, in order to obtain secondary normal distribution of rating data. All data are shown as mean ± standard error of the mean (SEM) if not otherwise indicated. Effects were statistically analysed for significance using paired or unpaired Student’s t-test and analysis of variance (ANOVA; Statistica 4.5, StatSoft Inc., Tulsa, OK, USA), p < 0.05 was considered significant.

### In vitro models

#### DRG cell culture

Acutely dissociated dorsal root ganglion (DRG) neurons were obtained as follows. Animals’ care was in accordance with institutional guidelines and approved by the appropriate authorities. Male adult Sprague–Dawley rats (4–8 weeks old, 280–350 g; Janvier, Le Genest-Saint-Isle, France) were deeply anesthetized with isoflurane (Forene^®^, Abbvie Deutschland, Wiesbaden, Germany), and rapidly decapitated. Spine was removed and chilled on ice in Dulbecco’s modified Eagle’s medium (DMEM) F12 medium (Sigma-Aldrich, Munich, Germany) containing 26 mM NaHCO_3_ (Roth, Karlsruhe, Germany), 100 IU ml^−1^ penicillin, 100 µg ml^−1^ streptomycin (Gibco, Darmstadt, Germany) and equilibrated with 95% O_2_ and 5% CO_2_. Neurons were incubated in collagenase CLS II (10 mg ml^−1^; Biochrome, Berlin, Germany) and accutase (1x; Sigma Aldrich) for 45–60 min at 37 °C, washed trice with medium, triturated with fire polished pasteur pipettes and resuspended in neurobasal medium (Invitrogen, Darmstadt, Germany) containing 2% horse serum (Gibco), B27 (Gibco), Pen/Strep (100 IU ml^−1^ penicilline, 100 µg ml^−1^ streptomycin; Gibco), 200 mM l-glutamine (Gibco) and NGF (50 ng ml^−1^; Gibco). Then they were plated on microscope cover slips (Ø 15 mm, transparent to 1470 nm laser; Roth), coated with laminin (1.5 µg/cover slip; Sigma-Aldrich). Finally, the neurons were stored in a humidified 5% CO_2_-atmosphere at 34 °C before being used within 24 h.

#### HEK293 cell culture

Human embryonic kidney (HEK293) cells were cultured in DMEM (Gibco) supplemented with 10% fetal calf serum (FCS Gold, Gibco), 100 IU ml^−1^ penicillin, 100 µg ml^−1^ streptomycin (Gibco) in a humidified 5% CO_2_-atmosphere at 37 °C. One day prior to transfection, cells (with a density of 100–400,000 cells/well in a 12-well plate) were seeded on poly-l-lysine-covered (10 µg ml^−1^, Sigma-Aldrich) microscope cover slips (Ø 15 mm) and transfected using 6 µl nanofectamine (PAA, Pasching, Austria), 1 µg rat TRPV1 for comparison with rat DRGs and 1 µg pTagGFP (100 µl per coverslip; cf. [37]) for fluorescent identification of transfected cells. Within 48 h after transfection cells were used for functional calcium imaging.

#### Calcium imaging

For measurements of free intracellular calcium ([Ca^2+^]_i_) the cells were loaded with 3 µM (HEK) or 1 µM (DRG) of the fluorescent calcium indicator FURA-2AM (1 mM in DMSO, Biotrend, Cologne; Germany) and 1 µl Pluronic F-127 (10% in DMSO, Calbiochem, Darmstadt, Germany) for 45–60 min (HEK) and 30 min (DRG) in Tyrode's solution containing 137.6 mM NaCl, 5.4 mM KCl, 0.5 mM MgCl_2_, 1.8 mM CaCl_2_, 10 mM HEPES and 5 mM glucose (adjusted to pH 7.3 with NaOH; Roth). After washing them trice with Tyrode’s solution and at least 20 min resting, cover slips were mounted in an open bath chamber (Series 40 Quick Change Imaging Chamber; Warner Instruments, Hamden, USA) on an inverted microscope (Olympus IX81; Olympus, Tokyo, Japan) equipped with an image acquisition and analysis system (xcellenceRT; Olympus) and superfused with Tyrode’s solution (~ 2 ml min^−1^). Cells were illuminated alternatingly with light of 340 and 380 nm wavelength (≥ 0.5 Hz) and the respective fluorescent signals at 510 nm were detected by an ORCA-R2 CCD camera (Hamamatsu Photonics, Hamamatsu, Japan; scheme see Fig. 1c). The ratio of fluorescence emission at 510 nm for excitation at 340 vs 380 nm excitation was used as relative change in [Ca^2+^]_i_ [22]. All experiments were carried out at room temperature.

#### Diode laser stimulation in vitro

A computer controlled near infrared laser (DL-1470, 1470 nm wavelength, max. power 10 W; Rapp OptoElectronic, Wedel, Germany) yielding effectively up to 460 mW output power, focused to a diameter of nominally 100 µm (Gaussian distribution, 1/e = 70 µm; objective UApo/340 20x/0.75, Olympus) was coupled into the microscope via an optical bench and a scanning system (UGA-40, Rapp; Fig. 1c). For adjustment of the laser, a CCD camera with an IR-sensitive InGgAs (photo)sensor (IR camera, Rapp; NI-IMAQ, National Instruments, Munich, Germany) was placed in a parallel optical port of the microscope and the system was aligned with the images obtained in calcium imaging. Hence, aiming at single or small groups of cells (depending on the cell density) on a microscope cover slip during one experiment was possible using the scanning system driven by SysCon software (Rapp). Stimulus intensity was changed by varying laser power (13–460 mW) and/or stimulus duration (1–300 ms).

**Fig. 3:**
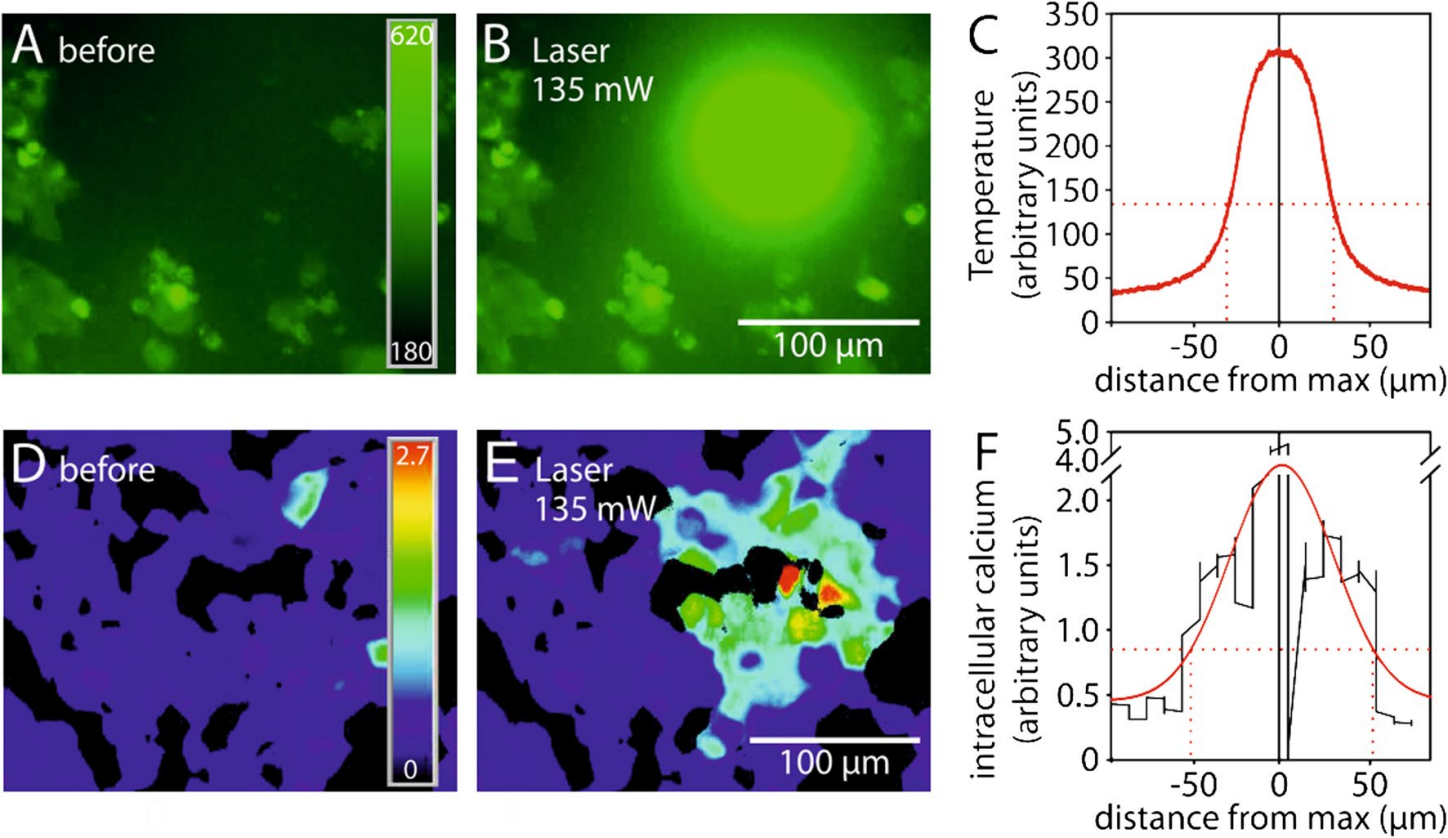
Focused diode laser stimuli rapidly increase temperature at the bottom of a cell culture dish. **A**–**C** Photothermal study using temperature-sensitive L-DNA beacons suspended in Tyrode's solution with GFP transfected HEK cells for size comparison. A laser pulse of 135 mW for 200 ms induces a fluorescence signal with a Gaussian spatial distribution (**C**). Dotted red lines indicate 1/e of maximum used to determine spot diameter (60 µm). **D**–**F** Ratiometric microfluorimetry of calcium transients in HEK cells transfected with TRPV1 and loaded with FURA-2. The calcium response profile of responding cells reflects the spatial heating pattern (**F**), dotted red lines indicate 1/e diameter. This diameter (about 100 µm) was used for definition of ROIs within laser radiated area

The laser used in cell physiology experiments in vitro was focused to the bottom of the cover slips, where DRG neurons or HEK cells had been plated. Stimulus location was observed via the infrared camera integrated in the microscope (Fig. 1c). Spot size of the area irradiated by IR-laser light as measured using this camera was 74 µm (1/e, inset in Fig. 1c). The size of the heated area was measured using thermosensitive molecular beacons that increase their fluorescence with increasing temperature (Fig. 3A–C; [29]). The spatial temperature profile was bell-shaped with a 1/e spot diameter of 61 µm. The spatial pattern of laser heat responses was determined using densely plated TRPV1 transfected HEK cells and showed an activated area of about 100 µm in diameter, again with a bell-shaped curve of intensities (Fig. 3D–F). Only cells within the laser heated area (i.e. 1/e radius according to the determined diameter) responded (Fig. 3D, E) indicating high spatial resolution. In conclusion, our laser system provides a precise means for stimulation of selected cells with high spatial and temporal resolution.

In order to characterize the heating pattern of the laser and taking the temporal resolution of the live cell imaging system into account, we applied stimuli of 200 ms in a suspension of 0.85 nM L-DNA thermosensitive molecular beacons [29] in Tyrode’s solution while measuring fluorescence intensity with the maximum possible acquisition frame rate of 10 Hz. Tachyphylaxis was tested with an ISI of 180 s. During the experiments over time spans of up to 45 min, no significant cell degradation attributable to diode laser irradiation was observed.

#### Chemicals

Capsaicin and capsazepine (CPZ; Sigma-Aldrich) were dissolved in ethanol and DMSO as stock solutions (100 mM and 10 mM), stored at 4 °C. Capsaicin, CPZ and ionomycin (1 mM stock solution in DMSO, Calbiochem) were dissolved in Tyrode’s solution to a final concentration of 10 µM shortly before each experiment. The drugs did not change the pH of the solutions by more than 0.1 and maximum ethanol concentration was 0.1‰.

#### Data analysis

Analysis of ratios of emission intensity at 340 nm/380 nm excitation and subtraction of background fluorescence were carried out off line with xcellenceRT software (Olympus). All cells within the 1/e diameter of the laser heated area (cf. Fig. 3F) were marked individually as regions of interest (ROIs; 12–150 per experiment), corresponding to the examined cells. The number of cells is given (n). Transfected HEK cells were considered TRPV1 positive only when responding to 10 µM capsaicin. DRG neurons were tested for viability and excitability by depolarisation using high potassium solution (140 mM KCl).

Relative change of fluorescence ratio was calculated by dividing the peak value by the preceding baseline. An increase of ≥ 23% was regarded as a significant response [18]. Thresholds were defined as the necessary stimulus duration at a certain fixed intensity or the necessary stimulus intensity at a certain fixed duration that induced a significant increase in intracellular calcium in 50% of the stimulated cells (T_50_ of sigmoidal fitted responder curve). All data are shown as mean ± standard error of the mean (SEM). Effects were statistically analysed for significance using two-tailed paired or unpaired Student`s t-test, p < 0.05 was considered significant.

## Results

### Characterization of laser stimuli

Extinction spectra of visible light and near infrared radiation showed the known absorption peak of water at 1450 nm (Fig. 1a, blue trace). DMEM F12 medium (light red) and neuronal cell suspension in DMEM F12 medium (dark red) displayed an additional peak around 550 nm, caused by the pH indicator phenol red. A parallel upward shift of the complete extinction spectrum was observed with suspended cells, due to scattering. In the near-infrared range, principally, scattering and absorption by biomolecules is by far exceeded by absorption in water [26]. As our data did not show any specific absorption bands of DRG neurons and absorption by water determines radiant heat energy deposition in nociceptive neurons and nerve terminals, absorption characteristics of neuronal cells can be considered the same as of water. Thus, wavelengths to heat neurons in the near IR ideally are near the absorption peaks of water. We therefore used near infrared laser stimuli of nearly identical wavelengths (1470 and 1475 nm; see enlarged inset in Fig. 1a), to stimulate human skin (Fig. 1b) and cells within the microscope (Fig. 1c).

When focused to 930 µm below human skin surface, laser stimuli rapidly heated nociceptive nerves within epidermis and dermis (Fig. 2a; up to 320 °C s^−1^; Fig. 2f) with temperature maxima at about 280 µm below surface (Fig. 2b, e). In addition to the intraepidermal nerve fibers that are evaluated clinically, there is also a dense network of dermal nerve fibers within this depth range (insert in Fig. 2a). Heat sensitive nociceptors are located between 20 and 570 µm below skin surface [54]. Peak temperature increase above baseline was 37.8 ± 0.5 °C (Fig. 2f) for strong suprathreshold stimulation (200 ms, 100 mW) reaching noxious temperatures within about 25 ms without any heat accumulation for interstimulus intervals (ISIs) ≥ 1 s. Baseline temperature in hands was 29.8 ± 0.5 °C, in feet 26.7 ± 1.2 °C. Temperature rapidly returned by passive cooling [time constant τ = 0.35 ± 0.01 s on skin, 0.29 ± 0.01 s on agar and 0.32 ± 0.01 s beneath surface (Fig. 2f–h)].

The laser stimulator embedded in the live-cell imaging microscope (diameter of irradiated spot about 74 µm) induced a bell-shaped spatial temperature profile with an 1/e spot diameter of 61 µm (inset in Fig. 1c) as characterized using thermosensitive molecular beacons (Fig. 3A–C; [29]). Using densely plated TRPV1 transfected HEK cells, laser heat responses were seen with a bell-shaped curve of response magnitudes within an area of about 100 µm in diameter, (Fig. 3D–F) but not outside, indicating high spatial resolution. In conclusion, our laser system provides a precise means for stimulation of selected cells with high spatial and temporal resolution.

### Diode laser heat pain in humans is encoded by TRPV1-expressing neurons

**Fig. 4:**
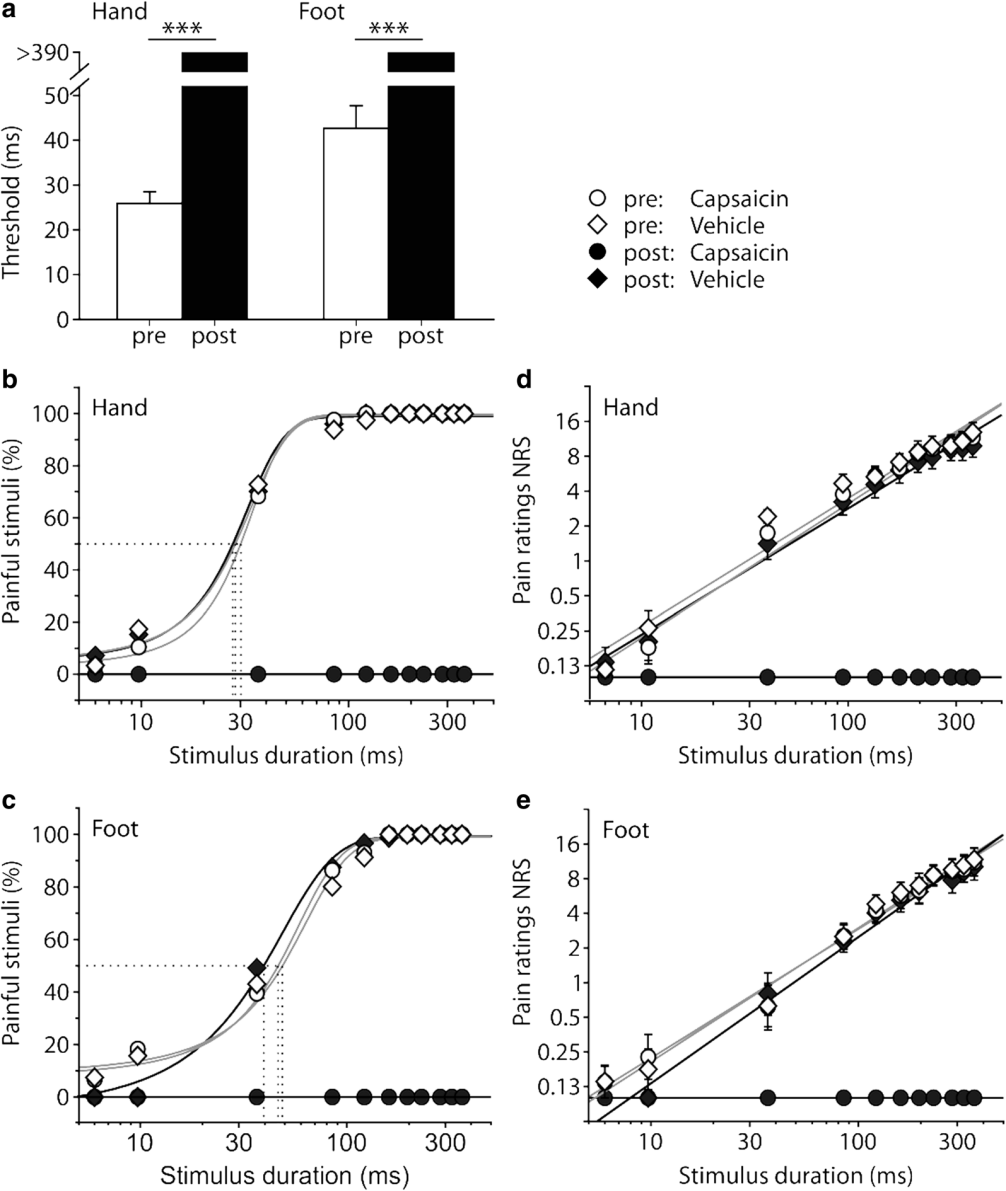
Human diode laser induced heat pain depends on TRPV1-expressing nerve fibers. **a** Mean pain thresholds (stimulus duration at 100 mW) on hand and foot (n = 10). After desensitization by 8% capsaicin for 24 h (Qutenza^®^), laser heat pain sensations were abolished and stimuli were not perceived even at maximum stimulus duration (390 ms) and laser power (100 mW); ***p < 0.001, paired t-test. **b**, **c** Psychometric functions (% painful stimuli) before (open symbols) and after (filled symbols) capsaicin (circles) or vehicle patch application (diamonds) for 24 h. T_50_ values indicated as dotted lines. **d**–**e** Pain ratings displayed a linear increase in double logarithmic coordinates following a power function with an exponent of 1.1 at the hand and 1.2 at the foot. Pain perception was completely abolished after TRPV1 desensitization by application of a capsaicin patch for 24 h (filled circles)

When stimulated with suprathreshold laser pulses, all human subjects (n = 10) reported a first clear, pricking pain sensation followed by a second more diffuse, burning pain, which is consistent with the activation of Aδ- and C-fibers. Pain thresholds at the hand (25.9 ± 2.6 ms at 100 mW laser power, 2.6 mJ, about 22.3 °C increase [from inset Fig. 2f]) were significantly lower than at the foot (42.6 ± 5.1 ms, 4.3 mJ, about 28.3 °C increase (Fig. 2f); p < 0.01, unpaired t-test, Fig. 4a; method of limits). In single subjects, stimuli were perceived as painful down to a minimal stimulation duration of 6 ms (0.6 mJ, about 7.6 °C increase [from Fig. 2f]). Incidence of painful trials increased with stimulus duration following sigmoidal functions (Fig. 4b, c); 50% thresholds (T_50_) were found to be 28.6 ± 0.8 ms (about 23.5 °C increase) at the hand and 43.7 ± 3.6 ms (about 28.6 °C increase) at the foot. Suprathreshold ratings showed a linear increase of perceived pain intensity in double logarithmic space with exponents of 1.1 to 1.2 (Fig. 4d, e; r^2^ of linear fits 0.97–0.99).

After complete desensitization of TRPV1-expressing skin nerve fibers by topical application of 8% capsaicin patch for 24 h (Qutenza^®^), laser induced heat pain was completely abolished (filled circles in Fig. 4d, e) and heat pain threshold was not detectable within the tested stimulus durations (up to 390 ms, 100 mW; p < 0.001 test vs. control area; see Fig. 4a). These results suggest that these laser pulses induced heat pain by activation of TRPV1-expressing neurons.

### Sensitivity to laser heat is co-expressed with capsaicin sensitivity in DRG neurons and TRPV1-expressing HEK cells

**Fig. 5:**
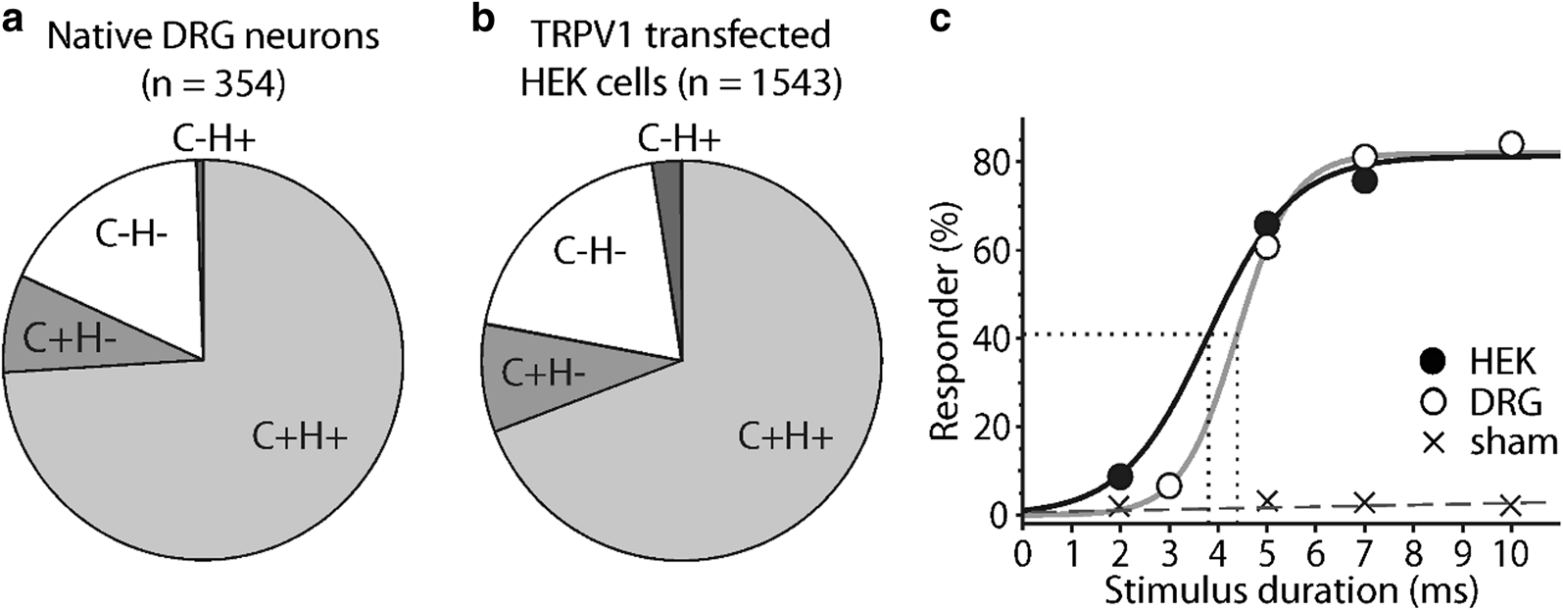
Diode laser heat sensitivity is associated with capsaicin sensitivity in DRG neurons and TRPV1 transfected HEK cells. **a** Response frequencies of DRG neurons and **b** TRPV1 transfected HEK cells. The vast majority of cells are either excited by both stimuli, capsaicin and laser heat (C+H+, light grey) or responsive to none (C−H−, white). **c** Responder rates of capsaicin sensitive DRG neurons (open) and TRPV1 transfected HEK cells (filled symbols) increase with increasing stimulation duration (135 mW). T_50_ of responding cells (dotted lines) were similar (DRG neurons 0.56 mJ, n = 115 and HEK cells 0.52 mJ, n = 213). Control data from sham-transfected HEK cells (only GFP transfected, n = 722; crosses) show hardly any responses (overall 3.4% responders)

Figure 5 shows cellular response patterns to laser heat and capsaicin stimulation of all cells at least once irradiated by laser (within 1/e diameter, see Fig. 2f). In 290 of 354 tested DRG neurons (82%) an increase of intracellular calcium ([Ca^2+^]_i_) in response to capsaicin (10 µM) was observed, indicating their native TRPV1-expression (Fig. 5a). Sensitivity to capsaicin and heat were significantly co-expressed: 262/290 (89%) of TRPV1-expressing neurons were heat sensitive and 262/264 (99%) heat sensitive neurons were capsaicin sensitive (Cohen’s Kappa 0.75, p < 0.001). It has to be noted that laser stimuli were of moderate intensity to avoid cell damage and cells at beam perimeter (within 1/e radius) were included in the analysis.

In TRPV1 transfected HEK cells, 1203 of 1543 (78%) were considered successfully transfected [increase of [Ca^2+^]_i_ when stimulated with capsaicin (10 µM)] (Fig. 5b). Of those, 1068 (89%) were also laser heat sensitive. Of the 1104 cells sensitive to heat 1068 (97%) were also sensitive to capsaicin (10 µM). Thus, sensitivity to heat and capsaicin were highly correlated (Cohen‘s Kappa 0.71, p < 0.001). Hence, expression of TRPV1 transfers laser heat sensitivity to non-excitable HEK cells. In cells only transfected with GFP but not TRPV1, a small fraction (25/722: 3.5%) did show a slight increase of [Ca^2+^]_i_ to laser heat stimuli (Fig. 5c).

Responder rates increased as a function of stimulus duration in a sigmoidal fashion in both cell types, like in the in vivo experiments in humans (Fig. 5c). The T_50_ responder threshold in HEK cells (3.8 ms at 135 mW, 0.52 mJ, n = 213) was slightly lower than in DRG neurons (4.2 ms at 135 mW, 0.56 mJ, n = 115), and the response magnitude higher at 5 ms stimulus duration (82% vs. 42% above baseline), but across all stimulus durations the response magnitudes did not differ significantly (ANOVA, main effect of cell type: F_(1,807)_ = 1.19, p = 0.28). Both cellular thresholds were markedly lower than T_50_ thresholds in humans at 100 mW (28.6 ± 0.8 ms at the hand), suggesting that substantial summation in the central nervous system (CNS) is necessary for laser heat pain perception.

### Encoding of the intensity of laser heat pulses by TRPV1 transfected HEK cells

**Fig. 6:**
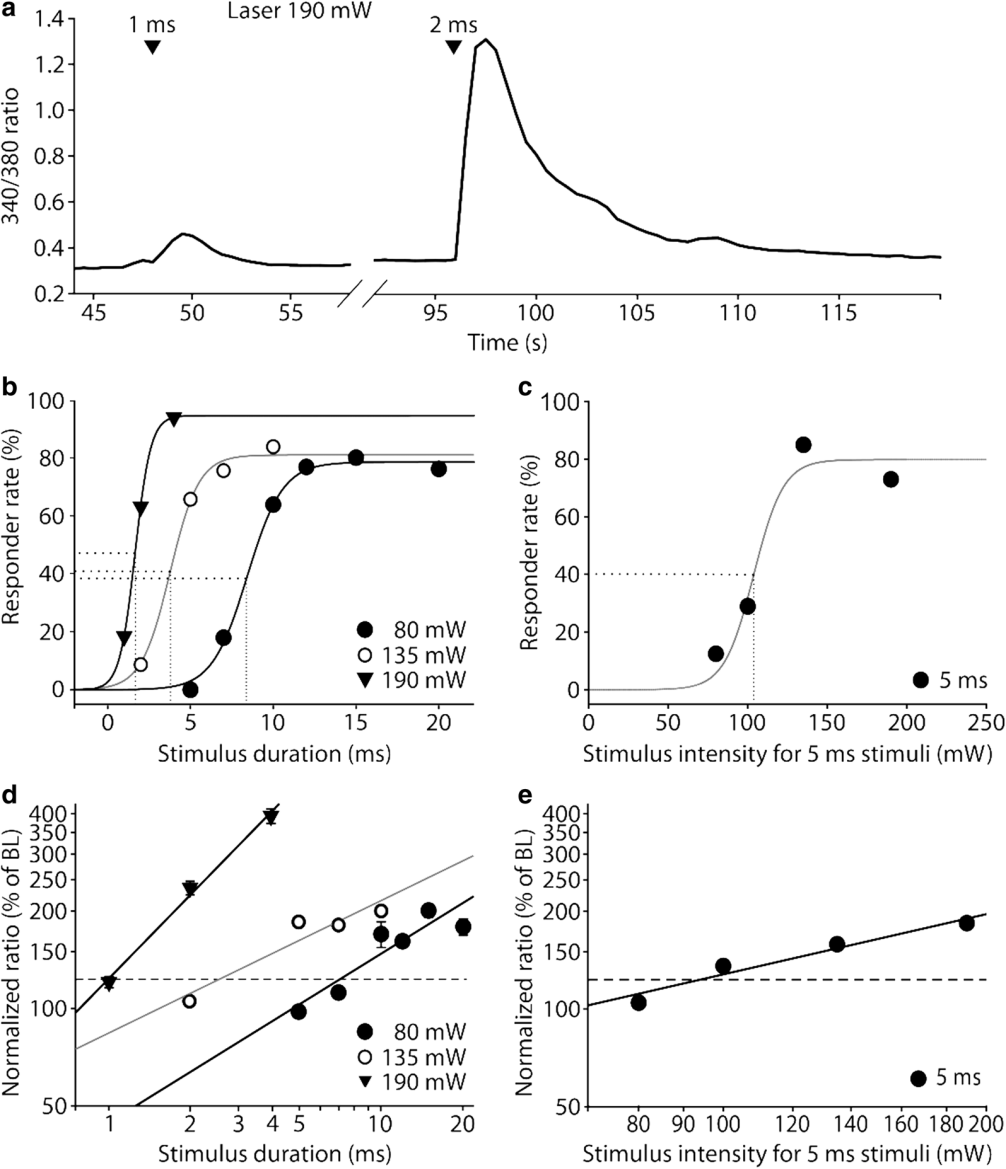
Diode laser heat induced calcium transients: stimulus-response characteristics of TRPV1 transfected HEK cells. **a** Original trace of a single HEK cell transiently transfected with TRPV1, which showed significant responses to 190 mW stimuli of 1 and 2 ms duration. **b** Responder rates of TRPV1 transfected HEK cells as a function of stimulus duration for different laser powers (80, 135, 190 mW; T_50_ responder thresholds given as dotted lines; n = 142–213). **c** At fixed stimulus duration of 5 ms the T_50_ was at 104 mW (n = 666, 0.52 mJ). **d** Calcium influx linearly increased with increasing stimulus duration at fixed laser power, with Stevens’ exponents between 0.40 and 0.85 (slopes). **e** Calcium influx linearly increased with increasing laser power at fixed duration, with a Stevens’ exponent of 0.62. Dashed lines indicate upper limit of non-specific heat-induced fluorescence artefacts [18]

Stimulus durations as short as 1 ms were sufficient to evoke significant responses in 17 out of 81 TRPV1 transfected HEK cells tested at 190 mW (21%, see Fig. 6a for a representative example). T_50_ threshold durations increased with decreasing laser power: 1.7 ms at 190 mW (0.32 mJ, n = 150), 3.8 ms at 135 mW (0.52 mJ, n = 213) and 8.4 ms at 80 mW (0.67 mJ, n = 142; Fig. 6b). Using a fixed stimulation duration of 5 ms, T_50_ threshold was 103.8 mW (0.52 mJ, n = 666; Fig. 6c). The efficacy for the induction of laser heat responses in TRPV1-expressing cells never reached 100% (Fig. 6b, c), possibly due to cells at the perimeter of the laser beam. In double-logarithmic scaling, stimulus-response functions were linear but seemed to show saturation at higher stimulus durations. At the highest laser power of 190 mW the Stevens’ exponent of 0.85 (slope) nearly matched those of our human pain perception data (exponents of 1.1 to 1.22, Fig. 4d, e). Those of lower laser powers (80 mW: 0.52, 135 mW: 0.4; Fig. 6d) and at a constant stimulus duration (5 ms: 0.62; Fig. 6e) were slightly smaller. We did not increase laser power beyond 190 mW, as we wanted to avoid cell damage and non-specific responses.

Saturation in calcium signals was seen earlier than in human pain psychophysics. This might be due to saturation of calcium influx from extracellular space: whereas fluorescence ratio at equilibrium with extracellular calcium (10 µM, ionomycin superfusion) reached 3.04 ± 1.34, calcium transients through TRPV1 (10 µM capsaicin as supramaximal dose) saturated at 1.54 ± 0.95 and the maximum laser heat-induced calcium transients were limited to 1.29 ± 0.61 (190 mW, 4 ms, n = 235, ± SD). These data suggest that transient opening of TRPV1 by heat pulses is nearly as efficient as TRPV1 activation for 60 s by a saturating concentration of capsaicin.

### Strength duration curves for heat thresholds of DRG neurons and TRPV1 transfected HEK cells

**Fig. 7:**
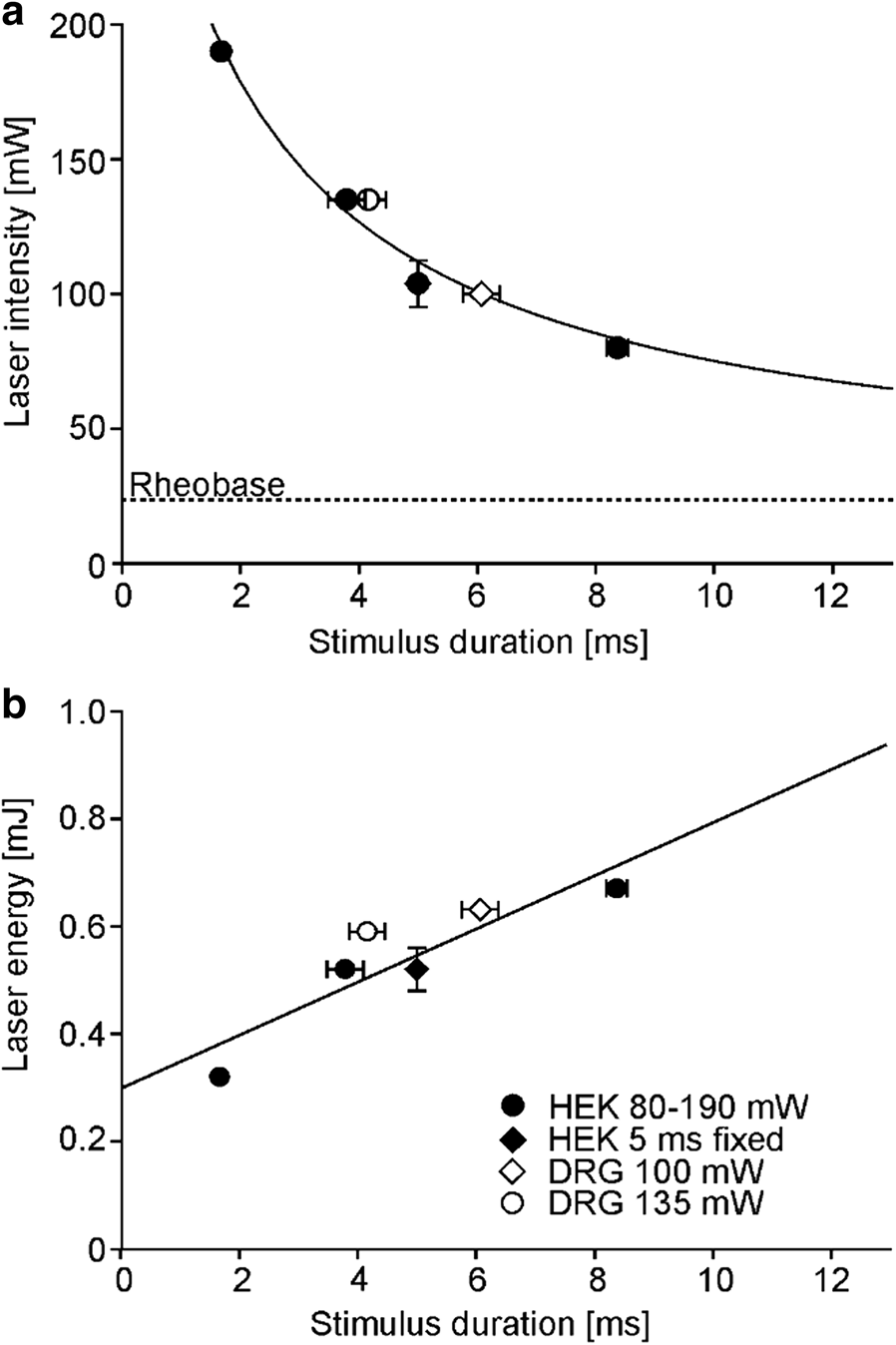
Strength duration relationships-comparison of thresholds in TRPV1 transfected HEK cells and DRG neurons. **a** 50% responder thresholds of DRG neurons (open) and transfected HEK cells (filled symbols) measured as laser power (mW) as a function of stimulus duration (ms). The hyperbolic fit suggests that thresholds may be expressed as a constant stimulus energy. The rheobase of the fitted function was calculated as 23.7 mW (dashed line). **b** 50% responder thresholds calculated as applied laser energy (mJ) plotted as a function of stimulus duration (ms). The rising linear function indicates loss of energy at the stimulation spot due to lateral thermal diffusion

Capsaicin-sensitive DRG neurons (open symbols, Fig. 7), displayed almost identical T_50_ values for laser heat stimulation as TRPV1-expressing HEKs (filled symbols, Fig. 7). Threshold values in both cell types concerning laser power and stimulus duration were fit by a hyperbola (R^2^ = 0.99, Fig. 7a) and displayed a rheobase of 23.7 mW indicating the minimum laser power for the induction of a laser heat response, and a chronaxia of 24 ms. In both cell types, the threshold energy rose with increasing stimulus durations (Fig. 7b), suggesting lateral diffusion of heat energy due to the small stimulus spot size as a confounding factor. These data suggest that for such short stimuli, over the whole range tested, TRPV1 is the defining receptor for thermal activation thresholds in both cell types.

### Tachyphylaxis in laser heat induced calcium transients in HEK and DRG neurons and in human heat pain

**Fig. 8:**
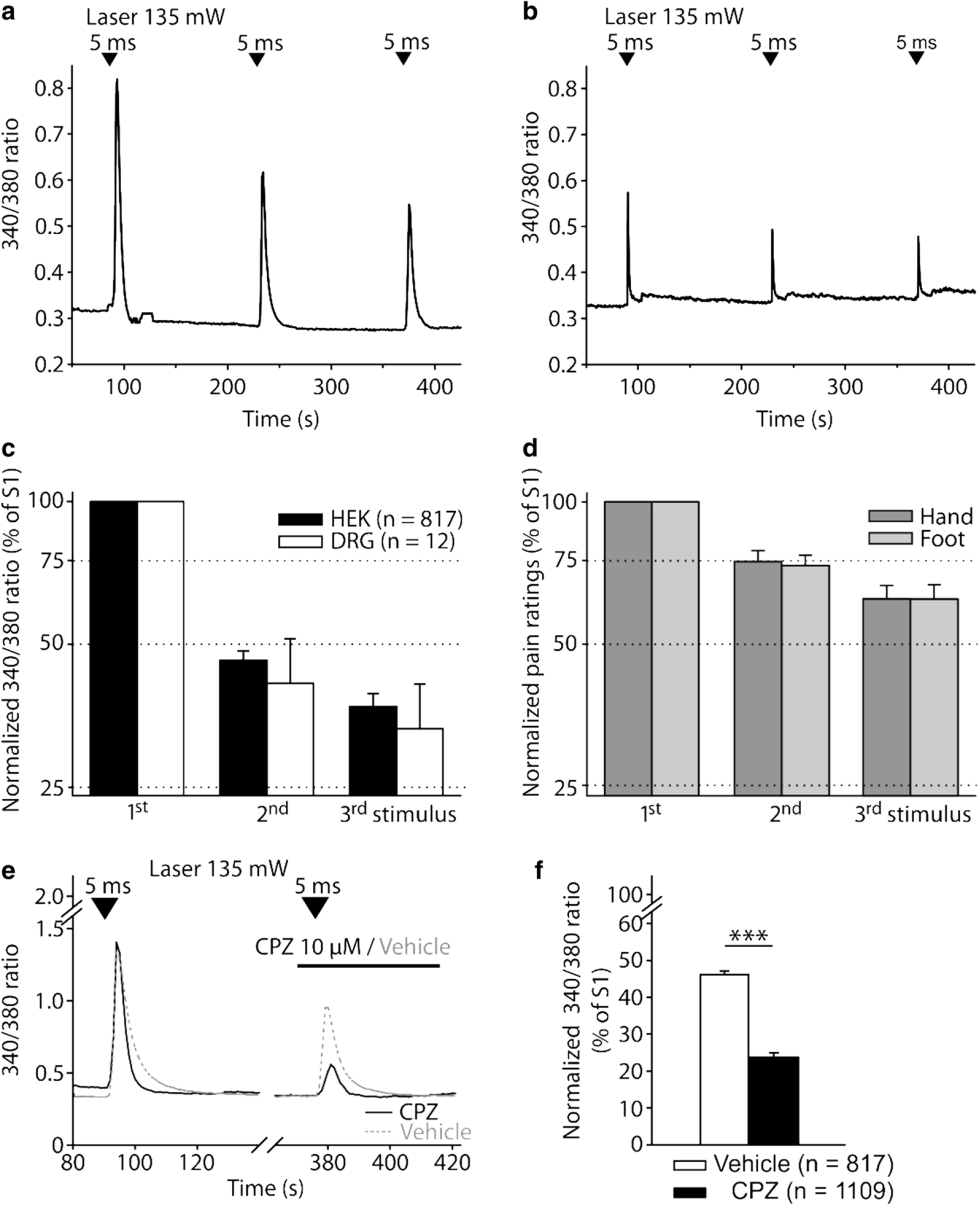
Tachyphylaxis of responses to repetitive diode laser heat stimulation in vivo as well as in vitro and partial block by TRPV1 antagonist capsazepine. **a** Original trace of responses of a single transfected HEK cell to repetitive diode laser heat stimuli (135 mW, 5 ms). **b** Original trace of responses of a single DRG neuron to repetitive diode laser heat stimuli (135 mW, 5 ms). **c** Tachyphylaxis of diode laser heat responses in HEK cells (filled bars) and DRG neurons (open bars) challenged three times with 135 mW, 5 ms laser stimuli (ISI 180 s). Changes in fluorescence ratio normalized to first stimulus. **d** Tachyphylaxis of diode laser induced heat pain on the human hand (dark grey) and foot (light grey) when stimulating the same spot repetitively (100 mW, 200 ms; ISI 180 s; n = 10). **e** Calcium transients of representative single transfected HEK cells challenged with repeated laser stimulation (135 mW, 5 ms) before (first stimulus) and during (second stimulus) incubation with 10 µM CPZ (black trace) or with vehicle (dashed grey). Values are given in 340/380 nm fluorescence ratio. **f** The competitive TRPV1 antagonist capsazepine (CPZ) significantly reduced laser heat responses compared to vehicle application (by 48.6%; ***p < 0.001, unpaired t-test)

When stimulated repetitively (5 ms, 135 mW) laser heat induced calcium transients were reduced from stimulus to stimulus in both cell types (TRPV1-expressing HEKs: second stimulus 46.3 ± 2.1% of first, third 37.0 ± 2.4% of first, n = 817; DRG neurons: 41.4 ± 10.0% and 33.2 ± 8.07%, n = 12; Fig. 8a–c); known as tachyphylaxis of heat-evoked neuronal signaling. Pain ratings decreased when stimulated repetitively (100 mW, 100 ms, n = 10), too, but to a significantly lesser degree (hand: second stimulus 75.1% (log − 0.120 ± 0.030) of first, third 62.8% (log − 0.190 ± 0.036); foot: 73.5% (log − 0.128 ± 0.030) and 62.8% (log − 0.188 ± 0.040; Fig. 8d). Control experiments with 297 sham transfected HEK cells (only GFP, but no TRPV1) showed no responses to repetitive heat (135 mW, 5 ms) nor to capsaicin (10 µM); assay sensitivity was verified by responsiveness to ionomycin (10 µM).

### Laser heat induced calcium transients are inhibited by the TRPV1 antagonist capsazepine (CPZ)

The selective TRPV1 antagonist CPZ (10 µM for 30 s) completely blocked calcium influx evoked by laser stimulation in half of all TRPV1 transfected HEK cells (562/1109). Remaining laser responses were significantly smaller than those after vehicle application, as CPZ reduced TRPV1 mediated laser Ca^2+^ transient amplitudes by 48.6% (response to second impulse 23.7% of first vs. 46.1% with vehicle; p < 0.001, unpaired t-test; Fig. 8f).

## Discussion

This study has shown that short diode laser heat stimuli (λ = 1470–1475 nm) induce a rapid temperature rise (up to 320 °C s^−1^) peaking at around 280 µm tissue depth in human skin, followed by exponential decay with time constants of about τ = 0.3 s. Similar temperature transients were induced in DRG and HEK cells inside a microscope. Geometry and wavelength of our diode laser setups enable comparative studies of human heat pain and signal transduction in nociceptive neurons at high temporal and spatial resolution. Bias through differences in wavelength (affecting absorption and penetration depth) or diameter (affecting passive cooling) was eliminated. Thus, intensity coding and tachyphylaxis can be studied across systems with nearly identical stimuli.

Laser heat pulses were painful for pulse durations above 6 ms (about 37.4 °C) with mean threshold energy of 2.6 mJ (52.1 °C) on hands and 4.3 mJ (55.0 °C) on feet. Desensitizing TRPV1-expressing skin nerve fibers with capsaicin abolished laser heat pain. TRPV1-expressing HEK cells responded to laser heat pulse durations above 1 ms with a mean threshold of 0.52 mJ (0.56 mJ in DRG neurons). Strength duration-curves in neurons and HEK cells indicated a rheobase of 24 mW, which likely indicates the extent of radial heat loss at equilibrium. HEK cell responses to laser heat were significantly reduced by TRPV1 antagonist CPZ.

Suprathreshold intensity coding was linear in humans across ≥ 1.5 orders of magnitude (in double logarithmic space), while intensity coding in transfected HEK cells started saturating within less than one order of magnitude. Higher thresholds and the wider dynamic range in human psychophysics are consistent with the need for temporal and spatial summation of peripheral nociceptive input in the CNS. Upon repetitive laser stimulation, tachyphylaxis was larger in cells than in human psychophysics, suggesting partial compensation by CNS-processing.

### Targeted thermal activation of nociceptive nerve endings in the skin in vivo

Free intraepidermal nerve endings are found up to the stratum lucidum, where they likely are the first line of sensors for potential or actual tissue damage. Using CO_2_ laser stimulation, intraepidermal nerve endings were found to be involved in peripheral encoding of noxious heat stimuli [6, 5, 46, 57, 58]. Functional estimates of the termination depth of nociceptive nerve endings indicate that they span a range from superficial epidermis to deep dermis (20–570 µm; [54]). From psychophysical and evoked potential thresholds, a mean effective depth of 130 µm has been estimated [53], which is within the subepidermal nerve fiber plexus. In human skin, both intraepidermal nerve fibers and subepidermal nerve fiber plexus express TRPV1 [42] and can be defunctionalized by high doses of topical capsaicin [3]. Deep nociceptors seem sufficient to explain heat sensitivity, since heat pain recovers more rapidly than intraepidermal nerve fiber density after topical capsaicin [50]. Our laser stimuli achieved maximum temperatures below the surface (280 µm, Fig. 2), likely heating directly the TRPV1 positive fibers of the subepidermal nerve plexus [54].

### TRPV1-expressing HEK cells as models of nociceptive DRG neurons

Calcium transients in HEK cells were generally larger than in DRG neurons, probably due to TRPV1 overexpression (10–20-fold above DRG, cf. [21] for TRPV3). In heat activated HEK cells, calcium flux through TRPV1 can be assumed to be the dominant source of intracellular calcium transients, whereas in DRG neurons calcium mainly flows through voltage gated calcium channels (75% [18]) that are activated by action potentials, elicited by depolarization of the neurons via TRPV1 [20]. In our data, threshold energies did not differ significantly between DRG neurons and HEK cells, the similarity in strength-duration curves in the range of 2–10 ms suggests no major differences in thermal gating kinetics of native rat TRPV1 in DRG neurons and heterologously expressing HEK cells.

### Intensity coding in heat nociception

Encoding noxious stimulus intensity is one of the criteria to identify a nociceptive neuron [1, 8]. The dynamic range of intensity coding in our data was larger for psychophysical thresholds than for cellular responses, which may be explained by spatial summation of input from a population of nociceptive afferents terminating at different depths below skin surface. Spatial summation of heat pain is well known for surface stimulation of the skin using thermodes [49]. Our data suggest that spatial summation also occurs between afferents terminating at different depths below surface; such populations of nociceptors will be sequentially recruited with increasing laser pulse energy. Recruitment of nociceptors with different thermal thresholds has been shown to contribute to the slowly increasing population response of type I AMHs (A-fiber mechano-heat nociceptor) during a prolonged burn injury [58], which in turn is thought to compensate for the pronounced adaptation of C-nociceptor response to the same stimulus [38] thus leading to a constant pain sensation.

### Peripheral pain memory: nociceptor fatigue and TRPV1 tachyphylaxis

According to the dual process theory [48], repetitive stimulation always induces inhibition (habituation) and facilitation (sensitization). Human heat pain displays rapid habituation reflecting peripheral fatigue when stimulation is restricted to the same nociceptive neurons [17]. Distinct tachyphylaxis to repetitive heat applied by either diode lasers or heated superfusion is known to occur in TRPV1-transfected HEK cells [55], generator currents as well as action potential discharges of neurons [20, 52] and nociceptive Aδ- and C-fibers in monkey and humans [2, 32, 45, 58]. Heterologous expression of TRPV1 inserted this type of pain memory into primarily non-excitable HEK cells. In our study, tachyphylaxis was even stronger in DRG neurons and TRPV1-expressing HEK cells than in human heat pain, probably reflecting “memory processes” distal to the first synapse of the nociceptive pathways that are partially compensated by CNS-processing. Tachyphylaxis of human heat pain, however, depends on ISI and was maximally expressed at an ISI of 10 s whereas reduction of heat-pain after 120 s (and 20 min) was about 55% (and 11%) of maximum [51]. Therefore, our paradigm (ISIs 180 s to account for recovery of calcium transients) underestimated maximum tachyphylaxis in human heat pain.

Upregulation and downregulation of TRPV1 tachyphylaxis are important regulators of nociception and pain sensitivity. For example, higher heat pain sensitivity of females has been related to reduced TRPV1 tachyphylaxis mediated by estradiol [44], whereas part of the analgesic efficacy of acetylsalicylic acid is related to its enhancing effects on TRPV1 tachyphylaxis [37].

### TRPV1 receptor involvement in heat pain

Our findings after defunctionalization of TRPV1 carrying nerve fibers in humans confirm their specific role in perception of phasic heat stimuli [10]. Application of high concentrated capsaicin patches (8%) > 10 h induces a complete lack of laser heat perception [24] and LEPs [50], while other sensory qualities are mostly preserved [30, 35, 36]. Similarly, pharmacological selective ablation of TRPV1 carrying nerve fibers leads to complete insensitivity to noxious heat in mice [11], rats [28] and swine [7]. However, TRPV1 knockout animals still show heat sensitivity [9, 13, 62], suggesting additional heat transducing mechanisms in the same neurons. It was recently proposed that TRPM3 and TRPA1 contribute to heat stimulus detection, and that the lack of each single thermosensor is compensated by another in single or double knock out animals [60]. The fact that almost all heat sensitive cells in our experiments were sensitive to capsaicin, but about 10% of capsaicin sensitive cells were not sensitive to heat may be explained by near-threshold laser intensity but far suprathreshold capsaicin dose (10 µM) used.

Our data indicate that TRPV1 carrying nerve fibers are necessary to transduce brief heat pulses in humans. TRPV1 is sufficient as the only excitable receptor in transfected HEK cells to detect brief laser heat pulses, and TRPV1 defines heat thresholds in rat DRG neurons and transfected HEK cells. Based on this, we suggest that non-TRPV1 heat transduction requires longer tonic stimuli and higher intensities, which are more likely to induce actual tissue damage. The role of TRPV1 in heat detection might thus be to indicate a threat of *possibl*e damage, while non-TRPV1 mediated heat detection is for *actual* damage.

### Limitations and technical considerations

The diode laser was applied directly, through the microscope cover slip, onto the DRG neurons or HEK cells. Previous studies had either stimulated from above through the medium with energy loss via absorption and diffraction [19] or used glass fiber optics to direct the laser radiation to the cells under study [27, 64]. Our system includes software-controlled aiming and allows stimulation of many different cells within one cover slip during the same experiment making a high cell throughput possible.

We used very small beam diameters enhancing radial heat loss and passive cooling. This allowed repetitive stimulation without heat accumulation but influenced the strength duration curve. Hyperbolic fit of strength duration curves in both, HEK cells and DRG neurons, suggests that laser pulse energy (power x duration, see Fig. 7) may be the relevant parameter for TRPV1 gating. However, unlike for large diameter CO_2_ laser stimulation of human skin [4] the threshold energies increased with increasing stimulus duration. Given the small heated volume, lateral diffusion is the most probable explanation [4]. Hence, when comparing studies and threshold values assessed with different types of laser stimulation the applied energy density (energy per area) and the diameter itself have to be taken into account. The Gaussian temperature profiles may have led to underestimation of heat sensitivity, because even for a conservative choice of beam radius (1/e), energy density at the perimeter is clearly lower than at the center and might thus not induce calcium influx.

## Conclusions and outlook

We directly compared cellular responses and human psychophysics for transient, non-damaging and punctate laser heat stimuli. We demonstrate that expression of TRPV1 fully conveys laser heat response properties observed in human heat pain and native sensory neurons to non-excitable HEK293 cells. The higher thresholds and wider dynamic range of intensity coding in humans suggests a spatial summation over a population of intraepidermal and subepidermal nociceptor populations. Our setup provides a useful mean to further study effects of transfection with other heat sensor candidates (TRPA1 or TRPM3) and their interaction with TRPV1.

## Data Availability

The datasets used and/or analysed during the current study are available from the corresponding author on reasonable request.

## Abbreviations

AMH: A-fiber mechano-heat nociceptor
CMH: C-fiber mechano-heat nociceptor
CNS: central nervous system
CPZ: capsazepine
DRG: dorsal root ganglion
HEK: human embryonic kidney (cells)
ISI: interstimulus interval
NRS: numeric rating scale
RTX: resiniferatoxin
T_50_: 50% responder threshold
TRPV1: Transient Receptor Potential Vanilloid 1
